# Association between Social Skills and Motor Skills in Individuals with Autism Spectrum Disorder: A Systematic Review

**DOI:** 10.3390/ejihpe10010022

**Published:** 2019-12-12

**Authors:** Reiko Ohara, Yuji Kanejima, Masahiro Kitamura, Kazuhiro P. Izawa

**Affiliations:** 1 Department of Health Science, Faculty of Medicine, Kobe University, Kobe 654-0142, Japan; 1473905m@stu.kobe-u.ac.jp; 2 Cardiovascular stroke Renal Project (CRP), Kobe 654-0142, Japan; 183k323k@stu.kobe-u.ac.jp (Y.K.); 136k902k@stu.kobe-u.ac.jp (M.K.); 3 Department of Rehabilitation, Kobe City Medical Center General Hospital, Kobe 650-0047, Japan; 4 Department of Public Health, Graduate School of Health Sciences, Kobe University, Kobe 654-0142, Japan; 5 Department of Physical Therapy, Kokura Rehabilitation College, Kitakyushu 800-0206, Japan

**Keywords:** autism spectrum disorder, restricted and repetitive behaviors, social skill, motor skill

## Abstract

Social communication and motor skill deficits are prevalent characteristics of individuals with autism spectrum disorder (ASD). This systematic research review investigates whether and how broad social skills and motor skills may be related among individuals with ASD. We performed a PubMed search of articles written in English, using these study inclusion criteria: (a) an association between social and motor and skills among individuals previously diagnosed with autism; (b) one or more social skills measures were used; and (c) one or more measures of gross or fine motor skills were used. We classified data into two categories, and we based the association of these variables on correlation coefficients, *p*-values, coefficients of determination, and authors’ description of “may be associated” and “may not be associated.” Despite heterogeneity among these relevant studies, a highly likely association between social and motor skills emerged. Of a total of 16 studies reviewed, 12 reported associations between these skill sets. Three studies reported that fine motor skills had a stronger relationship with social skills than did gross motor skills. Among the gross motor skills associated with social skills, object control skills seemed most closely linked to social skills. Among fine motor skills, manual dexterity seemed to most closely related to social skills.

## 1. Introduction

Autism spectrum disorder [ASD] is a neurodevelopmental disorder characterized by impairments in social communication and interaction and atypical patterns of restricted and repetitive behaviors [RRBs] [1]. Prevalence statistics suggest that about one in 59 children have ASD [2]. Various studies and experiment-based analyses have attempted to provide the probable causes of autism, including genetic variations, impairments in white matter connectivity and gray matter diffusivity, over-connectivity between neural assemblies, under-connectivity of functional brain regions, and environmental factors [3]. However, the exact causes are still unclear, and no commonly implemented diagnostic test or treatment is available for this disorder [4]. The diagnosis of autism at a very early age is challenging, due to the phenotypic and etiological heterogeneity of individuals with ASD [3]. Diagnosis is possible at 24 months, but it typically does not occur until 40 months of age [5]. Although both pharmacological and non-pharmacological interventions are available [6], the care is to support rather than fully treat this disorder. This means that there remains a continued search for more effective diagnostic and treatment methods.

Although not being included in diagnostic criteria, motor skill deficits are prevalent in individuals with ASD. Early descriptions of autism by Kanner [7] included characteristic limitations of the individual’s spontaneous activity and/or failures of the person’s body adjustment. More recently, a growing number of experts in ASD have reported the presence of diverse motor impairments, including clumsiness, motor coordination abnormalities, postural instability, and impaired gross and fine motor movements, relative to typically developing people [8]. Dewey et al. [9] and Green et al. [10] previously reported poor motor skill prevalence rates among children with ASD to be 59% and 79%, respectively. One of the reasons motor deficiency in individuals with ASD has received increasing attention is a growing awareness that this characteristic can potentially be used as a diagnostic criterion of this disorder [11]. 

In recent years, there has also been an increase in studies exploring the association between motor skills and social communication skills in the ASD population. Various studies have shown that motor skill deficits among individuals with ASD relate to their social communication functions. For instance, West [12] reported, in her meta-analysis, that infant motor skills and communication skills were related in ASD. MacDonald et al. [13] reported a relationship between motor skills and both adaptive social skills and adaptive communication skills. Similarly, Bhat et al. [14] reported that in infants at risk for ASD, early motor delays were predictive of the children’s future social communication delays. The authors recommended the surveillance of motor development from a very early stage of development, as motor delays become apparent at earlier ages than do social skill deficits. Any association between motor skills and social communication skills gains importance through the implication that early motor skill interventions might enhance later social development. Healy et al. [15] reported in their meta-analysis that early physical activity interventions seemed to have a moderately positive effect on later social functioning. Sam et al. [16] also reported that exercise mastery and social competence had a medium to large effect. Furthermore, Al Sagheer et al. [4] reported, within a well-acknowledged animal model of ASD, the valproic acid model, that motor dysfunction correlated with reduced social behavior. This finding suggested that early motor dysfunction might contribute to later social and communication deficits in a possible causal connection between these two skill sets. Although language development plays a key role in social development and may affect how ASD symptoms manifest, language impairments or social communication skill problems alone are not core ASD symptoms [17]. Gerbsbacher et al. [18] reported that delayed language development is a common but not a universal characteristic of autism since some studies that measured language ability did not show that language development among individuals with ASD differed from typical language development. 

Finding more specific relationships between motor and social skills may help in designing effective motor skill interventions for improved social functioning among individuals with ASD. Accordingly, this study seeks to systematically review available research to determine whether social skills and motor skills are associated in ASD and, if so, what specific motor skills may be especially related to social skills.

## 2. Materials and Methods 

### 2.1. Data Sources and Search Strategies

We searched for English articles published from May 1994 to August 2018 in the PubMed database, using combinations of search keywords that included “relat* OR associat*, motor, social, autism,” “link, balance OR postur* severity autism”, and “relat* OR associat* gait social autism.” We also conducted an MeSH search with the terms of “motor skill disorder” and “social skills”. As we were searching for studies of individuals diagnosed with ASD, we used a time period for our search that would yield articles published after the May 1994 publication of the American Psychiatric Association’s (APA) fourth edition of the Diagnostic and Statistical Manual IV [DSM-4].

### 2.2. Study Procedures

**Figure 1:**
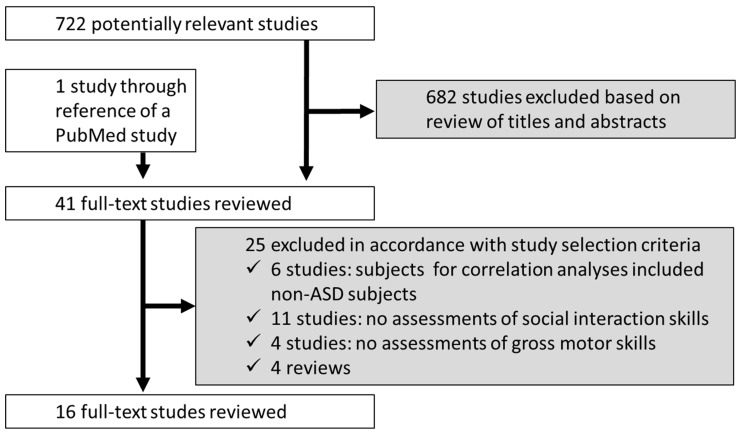
Study flow diagram.

Figure 1 shows the flow of study selection in this review. First, we read the titles and abstracts to select relevant studies examining associations between motor skills and social skills in persons with ASD. Second, we read in more detail those studies elicited by initial screening of these titles and abstracts to assess their contents. We then selected articles for this review if they met the following inclusion criteria: (a) the association between these variables of interest was examined within individuals previously diagnosed with autism including Asperger’s syndrome or “pervasive developmental disorders—not otherwise specified” as named under the DSM-4 diagnostic scheme so as to cover in this review individuals previously diagnosed as having ASD by more specific diagnostic criteria; (b) the study used one or more social skill measures: social skills included social interaction skills, social behavioral skills, verbal/nonverbal communication skills, and social comprehension skills, and the study used one or more social skills measures; (c) studies that included one or more measures of either gross motor skills or fine motor skills (gross motor skills use large muscle groups for coordinated body movements such as walking, running, jumping, and the maintenance of balance [19], whereas fine motor skills include grip strength, manual dexterity, and upper-limb coordination [20]); and (d) the studies were available in English. We excluded the literature if (a) the association between motor and social skills was examined among individuals with and without ASD (without separating them) and individuals who were at high risk for autism and/or typically developing [TD] individuals; (b) the studies used only language development measures; and (c) the studies assessed types of motor functions such as motor coordination.

### 2.3. Data Reduction and Analyses

We classified the data into two categories based on the correlation coefficients, *p*-values, coefficients of determination, or the authors’ descriptions that motor skills and social skills were or were not associated. The “may be associated” category includes studies in which the correlation coefficient (r) is higher than 0.2, and the *p*-value is lower than 0.05, η2 is higher than 0.1, or R2 is higher than 0.2. Studies were also included in the “may be associated” category if such analysis data were not provided but the author claimed that there was an association. The “may not be associated” category includes those studies in which the correlation coefficient (r) is lower than 0.2, and the *p*-value is higher than 0.05, η2 is lower than 0.1, or R2 is lower than 0.2. Studies were also included in the “may not be associated” category if such analysis data were not provided but the author claimed that there was little association. 

## 3. Results

### 3.1. Result of First and Second Screenings

Our search initially found 723 potentially relevant papers, of which 632 were excluded based on review of their titles and abstracts. The excluded articles included studies with a rat model or developmental disorders other than ASD. Of the 41 studies for which we read the full text, 25 were excluded based on the study’s inclusion/exclusion selection criteria, leaving 16 studies for this review (Figure 1).

### 3.2. Study Characteristics 

Table 1 summarizes each study, including its methods and results [13,21,22,23,24,25,26,27,28,29,30,31,32,33,34,35]. The following characteristics findings comprise the integrated data. We examined data from 16 studies that included 3355 individual participants with ASD. The studies’ participants’ ranged in age from 1 year to 39 years, with a mean age of 6.7 years. We included a wide age range so that we can generalize the associations in individuals with ASD. The ratio of male to female participants was 13%–87%. Only one study [21] was a longitudinal research design; all of the other studies were cross-sectional designs. 

### 3.3. Motor Skill Measurements 

Among the 16 studies reviewed, 8 measured both gross and fine motor skills, six measured only gross motor skills, and two measured only fine motor skills. Table 2 shows the methods used to measure motor skills, including the Movement Assessment Battery for Children-2 [MABC 2] [36], the Mullen Scales of Early Learning [MSEL] [37], the Vineland Adaptive Behavior Scale-Second Edition [VABS-2] [38], the Test of Gross Motor Development-2 [TGMD-2] [39], the Peabody Developmental Motor Scales Second Edition [PDMS2] [40], the Miller Function and Participation Scales [M-FUN] [41], the Learning Accomplishment Profile-Diagnostic [LAP-D] [42], and the Chinese Children Developmental Inventory [CCDI] [43]. Among these motor skill tests, the VABS-2 is a parent report test without direct observation, whereas the other tests assess performance through direct observation. The three studies using the VABS-2 may be potentially less accurate in motor skill measurement, as the accuracy of the VABS-2 is highly dependent on the honesty and objectivity of the parent respondents [22].

### 3.4. Social Skill Measurements 

Table 3 shows the methods used in these studies to measure social skills. The comprehensive measurement methods of social skills in these studies included the Social Skills Improvement System Rating Scales [SSIS] [44], Social Responsiveness Scale [SRS] [45], VABS-2, Autism Diagnostic Observation Schedule [ADOS] [46]/Autism Diagnostic Observation Schedule-2 [ADOS-2] [47], calibrated severity scores [mapped based on ADOS] [48], Autism Diagnostic Interview-Revised [ADI-R] [49], the Social Communication Questionnaire [SCQ] [50], the Learning Accomplishment Profile-Diagnostic [LAP-D] [43], the Devereux Early Childhood Assessment [DECA] [51], CCDI [43], and the Child Behavior Checklist/4–18 [CBCL] [52]. The ADOS/ADOS-2 [46,47], and the calibrated severity scores [48] assess social skills through observation, whereas the other tests are parent/caregiver questionnaires or interviews. The studies using parent/caregiver questionnaires or interviews may be less objective and/or accurate. Also of note, the SSIS [44], SRS [45], ADOS [46], calibrated severity scores [48], and ADI-R [49] contain assessment items pertaining to restricted and repetitive behavior.

### 3.5. Association between Motor Skills and Social Skills 

Twelve studies reported an association between motor skills and social skills in either overall test scores or subdomain test scores. More specifically, nine studies reported an association between overall motor skill scores and social skills. Three studies reported on both gross and fine motor skills (using the MABC-2), and associations with social skills in these studies varied with the social skill measurement methods used. Hannant et al. [23] found that the MABC-2 total motor skill scores were correlated with scores on the ADOS-2. The ADOS-2 is an in-person measure of ASD symptoms relying on a skilled examiner‘s observation, and the Hannant et al [23] study found a medium to large negative motor skill and social skill association effect (r = –0.647, *p* = 0.002). This study did not correlate motor skills with the adolescent version of the ADOS-2, the ADI-R, or the SCQ, both of which are parent-report/interview measures. Craig et al. [24] reported that MABC-2 total test scores correlated negatively with SCQ scores (r = –0.46, *p* < 0.001) in children with both ASD and a low intelligence score on an intelligence measure. Hirata et al. [25] used a different measurement method and found a moderate negative correlation between the MABC-2 total standard score and the SRS total score (r = –0.51).

Three other studies assessed both gross motor and fine motor skills and two of these reported that both gross and fine motor skills showed associations with social skills. In these studies, fine motor skills tended to have stronger correlations with social skills than did gross motor skills. Mody et al. [26] reported both gross motor and fine motor skills to be significantly associated with social interaction skills and also found that the scores for quality of social overtures were significantly associated with raw fine motor skill scores but not with raw gross motor skill scores. Hsu et al. [27] also reported that both gross and fine motor skills correlated with social skills, and their study reported a stronger correlation between social skills and fine motor skills than between social skills and gross motor skills (r = 0.767, *p* < 0.01 and r = 0.593, *p* < 0.01, respectively). MacDonald et al. [28] reported that fine motor skills and autism severity were significantly associated with adaptive social skills (β = 0.196, *p* < 0.05) as were fine motor skills and adaptive communicative skills (β = 0.358, *p* < 0.001) while there were no significant correlations in this study between social skills and gross motor skills.

Regarding studies of gross motor skills alone, Holloway et al. [29] assessed gross motor skills with two measurement tools (PDMS2 and M-FUN) and reported moderately high correlations between the overall (gross) motor quotient and the overall social skill score (PDMS2: r = 0.644, *p* < 0.01; M-FUN: r = 0.637, *p* < 0.01). Regarding studies of fine motor skills alone, Travers et al. [30] assessed fine motor skills, including handgrip strength and finger tapping speed, and found that both correlated with social skills, though grip strength correlated with social skills more strongly than did finger tapping speed (r = –.26, *p* = 0.045 and r = –.23, *p* = 0.07, respectively). Bishop-Fitzpatrick et al. [31] also assessed fine motor skills with finger tapping speed and manual dexterity, and these researchers found that only manual dexterity had a marginal correlation with social adaptive behavior (r = 0.20, *p* < 0.05).

Six studies reported an association between subdomains of gross motor functioning and social skills. Four of these included object control/aiming and catching skills. Holloway et al. [29] reported that stationary balance, locomotion, object manipulation, motor accuracy, and stability were moderately correlated with the overall social score. Motor planning and balance tended to be associated with the overall social score, but these findings were not statistically significant. Craig et al. [24] reported, in children with ASD and low intelligence, that MABC-2 aiming and catching scores correlated significantly with SCQ scores (r = –0.57, *p* < 0.001), while manual dexterity and balance were not correlated with SCQ scores. Third, Hirata et al. [25] reported that only the manual dexterity score correlated significantly with the SRS T-score (r = –0.70), whereas aiming, catching, and balance scores did not correlate with SCQ scores. Dadgar et al. [32] studied social skill subdomains and reported a significant correlation between the TGMD-2 object control subtest and the social skill of initiating joint attention (r = 0.838, *p* <.001), responding to joint attention (r = 0.831, *p* < 0.001), and initiating behavioral requests (r = 0.643, *p* = 0.002). MacDonald et al. [28] also reported that TGMD-2 object-control motor skills were significantly correlated with calibrated ASD severity, even while the total TGMD-2 score was not correlated with ASD severity. Neither object control nor locomotor skills correlated with SSIS scores. Travers et al. [33] reported that postural asymmetry during eyes closed and drift during two-legged standing with eyes closed were marginally correlated with the SRS score (r = 0.39, *p* = 0.07 and r = 0.35, *p* = 0.11, respectively). Postural waver during two-legged standing with eyes open was significantly correlated (r = 0.54, *p* = 0.01) with the SRS score. Among fine motor skills, manual dexterity tended to relate most to social skills.

### 3.6. Relationship between Motor Skills, Cognitive Skills, and Social Skills

Four reviewed studies found little association between these variables of interest. Colombo-Dougovito et al. [34] reported a very weak, non-significant association between social skills and gross motor abilities. Pusponegoro et al. [22] reported that, although low socialization scores were found in the children with ASD who had poor gross motor skills, there was no independent association between gross motor impairments and socialization skills. Kim et al. [21] conducted the only longitudinal study among the studies reviewed. They reported that neither gross motor nor fine motor skills significantly predicted improvements in social skills over time. They also examined the relationship between motor skills and cognitive skills and reported that motor skills predicted cognitive skills better than later social skills. MacDonald et al. [35] claimed an association between both gross and fine motor skills and calibrated autism severity scores, but the effect sizes in this study were considered too small for this review to accept, according to our a priori criteria (both effect sizes were represented by η2 = 0.02).

## 4. Discussion

This study examined possible relationships between motor skills and social skills in individuals with ASD over sixteen studies that met inclusion/exclusion criteria for inclusion in a systematic review. As twelve of these sixteen studies reported an association between motor skills and social skills, there is sufficient replication of this association to accept it as highly likely.

Among studies of gross motor skills, object control/aiming and catching skills, which include ball throwing, catching, and kicking, were found to be most likely to be related to social skills, based on repeated findings of these links in past research. Past studies have reported that lower-level object control/aiming and catching skills are prominent characteristics of individuals with ASD. Pusponegoro et al. [22] reported that impaired object control skills have been more often found among individuals with ASD than among typically developing individuals. Even when comparing children with ASD to those with intellectual disabilities (ID; i.e., an IQ score below 70), Craig et al. [24] reported that aiming and catching scores were lower in the ASD group than the ID group. They inferred that deficits on tasks involving the ability to integrate social and motor cues appeared to be specific to ASD. Our finding that object control/aiming and catching skills are associated with social skills in ASD populations of past research studies mirrors the Craig et al. [24] report that children with ASD have lower social skills than either groups of typically developing children or children with ID.

Among fine motor subdomains, our review found manual dexterity to be most likely to be related to social skills. McPhillips et al. [53] reported that children with ASD had a significantly lower score than children with specific language impairment for a threading task on the MABC-2, whereas other subtest scores of children with ASD were similar to these children with a language impairment. A threading task involves the coordination of both hands and a high level of visual-motor integration. Children with specific language impairment do not necessarily have social communication problems, but correlations between language impairments and motor impairments raise the possibility that the coordination of both hands relates to social skill deficits of individuals with ASD.

Our review of past research also suggests that the association between social skills and motor skills among individuals with ASD may be more significant for fine motor skills than for gross motor skills. This observation is consistent with West’s [12] findings of that fine motor skills were more strongly correlated than gross motor skills with communication/language skills. Not only in individuals with ASD but also in typically developing individuals, fine motor skills have been found to be more closely related to social skills more than gross motor skills. Kim et al. [21] reported that in typically developing children, fine motor skills were more strongly related to some cognitive and social skills than gross motor skills, and Davis et al. [54] suggested that an association between cognitive and motor subdomain skills was largely due to fine motor control and visual processing as opposed to gross motor functioning.

Although most of the studies we reviewed reported correlations between variables of primary interest in this review, four studies found little association, perhaps for several reasons. Firstly, none of these four studies examined motor subdomains. Some studies that found associations between motor skill subdomain scores and social skill scores but no association between overall motor skill scores and social scores. Thus, overall motor skill scores may not be sensitive enough to detect an association between these skills. Secondly, the two studies by Colombo-Dougovito et al. [34] and Pusponegoro et al. [22] used the VABS-2. The VABS-2 is a parent report test without direct observation for assessing social skills, and the absence of direct observation may lead to less accurate measurements of social deficits. Thirdly, there was only one longitudinal study [21] in our review, perhaps limiting the detection of correlations or even a possible directional relationship between these variables.

### 4.1. Underlying Mechanisms to Explain Motor and Social Skill Associations

While explanations for the apparent correlation between motor skills and social skills among individuals with ASD remain unclear, a possible mechanism includes shared neuroanatomical functioning between these skill sets. Among individuals with ASD, postmortem and brain imaging studies have consistently identified the cerebellum as one of the most abnormal brain regions associated with ASD, specifically including loss of cerebellar Purkinje cells in this population [55,56]. The cerebellum is considered critical for motor coordination and movement control, and but it is also implicated in higher functions such as cognition, speech, and emotion, all of which relate to social interaction [4]. The cerebellum controls motor balance and timing and has an important contribution in facilitating language and executive functions, all behaviors associated with social skills in individuals with ASD [57]. The cerebellum is also crucial for sensory-motor integration, with deficits in motor coordination and visual feedback control linked to impaired cerebellar functioning [58,59]. Because perception and action form an integrated loop [60], atypical visual processing may also contribute to the motor disturbances in ASD [8], perhaps influencing social interaction in turn [61].

We found that object control skills and manual dexterity are most closely related to social skills among studies of individuals with ASD. As both object control skills and manual dexterity require visual–motor integration, a link between visual–motor integration and social skills in individuals with ASD may evolve through maturation. 

In addition, Rourke [62] elucidated the difference between language attainment and social skill development in ASD. He reported that the acquisition of language may be prevented by substantial destruction or permanent disruption of white matter within the left cerebral hemisphere. He suggested that, in ASD, there may be a general condition of white-matter deterioration rather than the one confined to the left hemisphere; this is why it is important to examine the associations between motor skills and broad social skills other than language development.

### 4.2. Developmental Perspectives

The development of motor skills and social skills are reciprocally intertwined. Social skill development involves learning complex motor sequences [63], including nonverbal communications that require relevant eye contact, gestures, and other social synchronization. Core impairments in interpreting others’ social cues may, in turn, play a role in limiting motor learning among individuals with ASD [64], since many motor skills are learned by watching others [30]. Moreover, movement is a fundamental aspect of children’s play, and early motor delays and clumsiness may limit the social opportunities of young children, reducing opportunities to practice both social cognition and motor skills.

### 4.3. Clinical Importance

As correlations between motor skills and social skills were observed in most of the studies we reviewed, motor skill deficits may predict social impairments in individuals with ASD (and the reverse prediction may also apply.) To use motor skills as diagnostic clues, further research using longitudinal designs will be needed to determine if there is a causal relationship between these skill sets. If motor skill deficits were found to precede social skill deficits, early motor delays might assist the early diagnosis of ASD, and interventions for motor skill impairments might be beneficial in improving later social skills problems of individuals with ASD. Interventions for object control skills and dexterity might be of particular help in improve social skills. Further research might address the types of activities/exercises that may be most effective for improving social skills in this population.

### 4.4. Study Strengths and Limitations 

This is the first systematic review, to our knowledge, of the association between social skills, including but not limited to language skills and motor skills. This is also the first review to investigate the association between motor subdomain scores and social skill scores in greater detail. However, we could couldno’t make sufficient effort (such as inclusion and exclusion criteria, methods of statistical analysis) to normalize/standardize measurements of motor skills and social skills before drawing results and conclusions. Even among those studies we were able to review, measurement methods for motor skills and social skills varied. The subdomains and evaluation methods were different in each standardized test, challenging the ability to summarize results across studies. For example, the ADOS may not be sensitive enough to measure all of the social skills in individuals with ASD [34]. Maddox et al. [65] reported that social communication difficulties measured by the ADOS-2 are not specific to ASD, particularly in clinically complex settings. In addition, some social skill tests contained a repetitive and restricted behaviors subdomain that may have contributed to motor skills correlations, partially confounding the presumed independence of these social and motor skill measurements. 

We permitted standardized social skill tests that included restricted and repeated behavior (RRB) domains because RRB domains in the standardized social skill tests were not separable from other domains measured by the tests. Of note, however, RRBs do not necessarily refer to social skills, as they include (i) stereotyped or repetitive motor movements, use of objects, or speech, (ii) insistence on sameness, inflexible adherence to routines, or ritualized patterns and/or verbal/nonverbal communication, and (iii) highly restricted, fixated interests that are abnormal in intensity or focus by definition (American Psychiatric Association, 2013) [1]. The data analysis methods also varied across these studies, and we could not conduct a meta-analysis because there was insufficient raw data available. Additionally, there were differences in the ages of the individuals with ASD in these studies. As the development stage differs among ages, it may not have been accurate enough to include all ages. In addition, assessing social skills of very young children with ASD can be especially challenging as their social communication skills are even more limited than those of adults with ASD [66]. Finally, only one of the studies we reviewed used a longitudinal (versus a cross-sectional) research design, limiting any inferences about a causal relationship between these variables, as discussed above. 

## 5. Conclusions

In the context of this small sample of studies to review and the important differences between them in their measurement methods and research designs, our conclusions should be considered preliminary and tentative until further research has provided further clarification regarding the relationship between motor skills and social skills in individuals with ASD.

## Figures and Tables

**Table 1:** Summaries of the present study.

Author (Year)	Title	ASD Participants	Motor Tests	Social Tests	Other Tests	Results
Associated						
MacDonald M. et al. (2013) [13]	The relationship of motor skills and adaptive behavior skills in young children with autism spectrum disorders	ASD194 children (157 M, 37 F) aged 14-49 monthsnon-ASD (DD)39 children (M 27 F 12) in the same age range	•Mullen scales of early learning •gross motor skills •fine motor skills	The Vineland Adaptive Behavior Scales, 2nd Ed.		•Fine motor skills and calibrated autism severity were significant predictors of adapted behavior composite, daily living skills, adaptive social skills, and adaptive communicative skills. •Gross motor skills as predictors of daily living skills.
Kim H. et al. (2016) [21]	Relations among motor, social, and cognitive skills in pre-kindergarten children with developmental disabilities	ASD •373 children, ages between 3.55 and 5.70 years old	•Learning Accomplishment Profile-Diagnostic •gross motor •body movement •object movement •fine motor •manipulation •writing	•Devereux Early Childhood Assessment •initiative •self-control •responses	•Learning Accomplishment Profile-Diagnostic •cognitive •language •naming •comprehension	•Neither gross motor nor fine motor skills significantly predicted improvements in their cognitive skills or social skills.
Pusponegoro H.D. et al (2016) [22]	Gross Motor Profile and Its Association with Socialization Skills in Children with Autism Spectrum Disorders.	ASD •40 children aged between 18 months and 6 years TD •40 children aged 18 months-6 years	•Vineland Adaptive Behavior Scales, 2nd Edition; Gross motor subdomain	•Vineland-II; Socialization subdomain		•Low socialization scores in the children with ASD who had poor gross motor skills. •Did not find an independent association between gross motor impairments and socialization skills.
Hannant P. et al. (2016) [23]	Sensorimotor Difficulties Are Associated with the Severity of Autism Spectrum Conditions	ASD 18 children (13 M, 5 F) aged 7–16 years TD 18 children (7 M, 11 F) aged 6–12 years	•The Movement Assessment Battery for Children—2 •manual dexterity •ball skills •static and dynamic balance •The Beery-Buktenica Developmental Test of Visual-Motor Integration, Sixth Edition •visual motor integration •visual perception •fine motor coordination	•British Picture Vocabulary Scale-Third Edition •receptive language •The Social Communication Questionnaire (Lifetime form) •Autism Diagnostic Observation Schedule-2nd Edition •Autism Diagnostic Interview-Revised	•Sensory Profile •sensory responsivity •modulation •behavioral emotional response •Wechsler Abbreviated Scale of Intelligence-2nd Edition •verbal and non-verbal intelligence	•The MABC2 significantly correlated with the ADOS-2 with medium to large effect. •The MABC2 did not correlate with the ADI-R or SCQ.
Craig F. et al. (2018) [24]	Motor competency and social communication skills in preschool children with autism spectrum disorder.	ASD + ID 46 children between 3-6 years ID 42 children aged 3–6 years	•Movement Assessment Battery for Children-second edition (MABC-2) •manual dexterity •aiming and catching •balance	•Social Communication Questionnaire	•Leiter International Performances Scale Revised (Leiter-R) —IQ level	•In the ASD+ID group, a significant negative correlation between MABC-2 aiming and catching scores with SCQ scores. •MABC-2 total test scores correlated negatively with SCQ scores.
Hirata S. et al. (2014) [25]	Relationship between motor skill and social impairment in children with autism spectrum disorders	ASD 26 children aged 7–16 years with the IQ between 73-124	•Movement Assessment Battery for Children-second edition (MABC-2) •manual dexterity •aiming and catching •balance	•Social Responsiveness Scale		•The manual dexterity standard score was significantly correlated negatively with the SRS T-score. •The correlation between the MABC-2 total standard score and SRS total score was moderately high, but it did not reach the significance level.
Mody M. et al. (2017) [26]	Communication Deficits and the Motor System: Exploring Patterns of Associations in Autism Spectrum Disorder	ASD 1781 children aged 2–15.5 years, with all IQ levels (gender composition not specified)	•Vineland Adaptive Behavior Scale-Second Edition(VABS); Gross motor subdomains •Mullen Scales of Early Learning; Fine motor scales	•Mullen Scales of Early Learning -expressive/receptive languages •VABS; Interpersonal subdominant •Autism Diagnostic Observation Schedule -quality of social overtures		•Both GM and FM skills were found to be significantly associated with social interaction skills. •There was a significant negative association between QSOV scores and fine motor raw scores but not gross motor raw scores. •There was no significant association of QSOV scores with gross motor skills.
Hsu H.C. et al. (2004) [27]	The relationship of social function with motor and speech functions in children with autism	ASD 32 children (27 M, 5 F) (mean age, 44.5 months) (mean age, 44.9 months)	Chinese Children Developmental Inventory •Gross Motor •Fine Motor	Chinese Children Developmental Inventory •expressive language •concept comprehension •social comprehension •personal social		•Pearson’s correlation coefficient showing that all achieved statistical significance. •Using a stepwise linear regression test, the results showed the PS function was highly correlated with SC. •The remaining variables of GM, FM, EL, and CC did not significantly increase the association.
MacDonald M. et al. (2013) [28]	Motor Skills and Social Communicative Skills in School-Aged Children With Autism Spectrum Disorder	ASD 23 high-functioning and 12 PDD-NOS, aged 6–15 years old with the IQ above 64	•Test of Gross Motor Development-2 •locomotor skills •object control skills	•Social Skills Improvement System Rating Scales •Calibrated ASD severity score		•Total raw motor skill scores were not a significant predictor of calibrated ASD severity holding all other variables in this model constant. •Object-control motor skills significantly predicted calibrated ASD severity. •Neither the locomotor or object-control subscale predicted standardized social skills.
Holloway J.M. et al. (2018) [29]	Relationships Between Gross Motor Skills and Social Function in Young Boys With Autism Spectrum Disorder	ASD 21 boys aged 48–71 months, previously diagnosed as having ASD	•Peabody Developmental Motor Scales Second Edition (PDMS2) -gross motor skills •stationary •locomotion •object manipulation •Miller Function and Participation Scales (M-FUN) -gross motor skills •motor accuracy •motor planning •stability •balance and equilibrium •weight shifting	•The Social Skills Improvement System Rating Scales(SSIS) •social skills •problem behaviors		•Moderately high correlations were found between the overall PDMS2 motor quotient and the overall SSIS score. •Moderately high correlations were also found between the M-FUN motor score and the overall SSIS score. •There were moderate, positive correlations between the overall motor score and the communication, assertion, empathy, and engagement subtests. •No significant relationships were found between overall motor and SSIS problem behaviors. •All subtests of the PDMS-2 had positive, moderate correlations with the overall social score. Motor accuracy and stability had moderate, negative correlations with overall social scores.
Travers B.G. et al. (2015) [30]	Brainstem White Matter Predicts Individual Differences in Manual Motor Difficulties and Symptom Severity in Autism.	ASD 67 males aged 5–39 years TD 42 males aged 5–39 years	Fine motor •grip strength •finger tapping speed	•Social Responsiveness Scale	IQ - Differential Abilities Scales, WISC-III, WAIS-III	•Weaker grip strength was significantly correlated with more severe autism symptoms in the ASD group, and slower finger tapping was marginally correlated with more severe autistic traits in the ASD group.
Bishop-Fitzpatrick L. (2017) [31]	Correlates of Social Functioning in Autism Spectrum Disorder: The Role of Social Cognition	ASD 108 individuals aged 9–27.5 years old with normal IQ	Fine motor skills •speed - finger tapping for the dominant hand •manipulative - grooved pegboard test for the dominant hand	•Vineland Adaptive Behavior Scales •Socialization domain •Child Behavior Checklist/4–18 •Social Problems •does not get along with other kids •gets teased a lot	Social cognition (Theory of Mind) •Sally-Anne experiment •first-order false-belief task •John-Mary experiment •Peter-Jane experiment •second-order false-belief task	•Greater social cognition, but not motor function, was significantly associated with better social functioning when controlling for sex, age, and intelligence quotient.
Dadgar H. et al. (2017) [32]	The Relationship between Motor, Imitation, and Early Social Communication Skills in Children with Autism.	ASD 20 children aged 3–5 years old	•Test of Gross Motor Development-2 •locomotor skills •object control skills	•Early social communication scale (ESCS) •initiating joint attention (IJA) •responding to joint attention (RJA) •initiating behavioral requests (IBR) •responding to behavioral requests (RBR) •initiating social interaction (ISI) •responding to social interaction (RSI)	•Motor Imitation Scale	•A significant correlation was obtained between TGMD-2 object control subtest with IJA, RJA, and IBR, but not with RBR, ISI, and RSI. •The correlation between TGMD-2 locomotor subtests with ESCS subscales was not significant.
Travers B.G. et al. (2013) [33]	Motor difficulties in Autism Spectrum Disorder: Linking Symptom Severity and Postural Stability	ASD •26 adolescents and adults (24 M, 2 F) aged between 16 years 8 months to 28 years 10 months TD •26 participants (24 M, 2 F) aged between 18 years 2 months to 30 years 10 months with an IQ of 80 and over	•Nintendo® Wii balance board •Postural Stability •Visual input condition (eyes open or eyes closed), and the number of legs used (one or two).	•Social Responsiveness Scale •Repetitive Behavior Scale-Revised	•Wechsler Abbreviated Scale of Intelligence-IQ	•The SRS demonstrated a small-sized, non-significant correlation with postural asymmetry with eyes opened, but a medium-sized, marginally significant correlation with postural asymmetry during eyes closed. •There was a trend for more asymmetrical standing to be associated with greater social symptom severity, but this relation did not reach significance.
Colombo-Dougovito A.M. et al. (2017) [34]	Exploring the interaction of motor and social skills with autism severity using the SFARI Dataset	444 children with autism (more severe) and 39 with ASD (less severe) aged between 48–150 months.	•Vineland Adaptive Behavior Scales, 2nd Edition; Gross motor subdomains	•Social Responsiveness Scale		•Effect sizes further demonstrate a very weak association between gross motor and social skills and group severity. •The association between social skills and gross motor abilities revealed as very weak and nonsignificant.
MacDonald M. et al. (2014) [35]	Motor skills and calibrated autism severity in young children with autism spectrum disorder.	ASD 136 children aged 14 (12)–33 months non-ASD (DD) 23 children in the same age range	•Mullen scales of early learning •gross motor skills •fine motor skills	•Calibrated Autism severity scores		•Gross motor skills were related to calibrated autism severity. •Fine motor skills were related to calibrated autism severity. •Effect sizes were small for both fine and gross motor skills.

ASD, autism spectrum disorder; TD, typically developing; ID, intellectual disabilities.

**Table 2:** Measurements of the motor skill tests.

Author (Year)	Test Name	Age Coverage	Test Description	Procedure
Kim H. et al. (2016) [21]	Learning Accomplishment Profile-Diagnostic (LAP-D)	36 to 72 months	•Gross motor (57 items) - body movement, object movement •Fine motor (59 items) - manipulation, writing	The subject is asked to perform simple actions.
Hannant P. et al. (2016) [23]	The Movement Assessment Battery for Children—2 (MABC 2)	3 years to 16 years 11 months	•Gross motor - ball skills (2 items), e.g., catching, throwing - balance (3 items) e.g., static and dynamic balance •Fine motor - manual dexterity (3 items), e.g., a unimanual or bimanual task, an untimed drawing task	•Quantitive assessment of the performance e.g., time taken, the number of successful executions.
Craig F. et al. (2018) [24]				
Hirata S. et al. (2014) [25]				
MacDonald M. et al. (2013) [13]	Mullen scales of early learning (MSEL)	•Gross Motor: birth to 33 months •Fine Motor: birth to 68 months	•Gross motor (35 items) - central motor control - mobility in supine, prone, sitting, and upright positions •Fine motor (30 items) - motor planning and control - unilateral and bilateral manipulation - writing readiness	•Majority of items are scored as either 1 (present) or 0 (not present/completed). •Standarized scores do not provide subscores below 20.
MacDonald M. et al. (2014) [35]				
Mody M. et al. (2017) [26]				
Mody M. et al. (2017) [26]	Vineland Adaptive Behavior Scale-Second Edition (VABS-2)	Birth to 90 years with disabilities	•Gross motor - sitting, beginning mobility, beginning to stand and walk, throwing a ball, climbing, running, using stairs, jumping, hopping, skipping, walking places, catching a ball, riding a tricycle or bicycle, lifting and carrying, stamina	•Item scores were obtained by reports from parents or teachers, through item ratings of “never”, “sometimes”, “usually performed”, "don’t know", or “no opportunity”.
Colombo-Dougovito A.M. et al. (2017) [34]				
Pusponegoro H.D. et al. (2016) [22]				
Hsu H.C. et al. (2004) [27]	Chinese Children Developmental Inventory (CCDI)	6 to 78 months	•Gross motor •Fine motor	
MacDonald M. et al. (2013) [28]	Test of Gross Motor Development-2 (TGMD-2)	3 to 10 years	•Gross motor -locomotor skills, e.g., run, gallop, leap, horizontal jump, hop, and slide - object control skills, e.g., striking a stationary ball, stationary dribble, kick, catch, overhand throw, and underhand throw	•Require the subject to perform threetrials of each skill. •Each skill is evaluated based on 3 to 5 performance criteria. •If two out of three trials are performed correctly, score 1, and if not, score 0. •Scores for locomotor and object control skills are obtained by summing the scores for related skills. •Total scores range from 0 to 48.
Dadger H. et al. (2017) [32]				
Holloway J.M. et al. (2018) [29]	The Peabody Developmental Motor Scales Second Edition (PDMS2)	Birth to 5 years	•Gross motor skills -stationary, e.g., standing on a foot or reaching on tiptoes -locomotion, e.g., walking, running, and jumping. - object manipulation, e.g., throwing, catching, and kicking a ball	•Participants are requested to perform as instructed.
Holloway J.M. et al. (2018) [29]	The Miller Function and Participation Scales (M-FUN)	2 to 7 years	•Gross motor skills - motor accuracy, e.g., kick or jump correctly -motor planning e.g., planning and performing tasks in a smooth, coordinated manner -stability e.g., maintaining upright posture while performing tasks -balance and equilibrium e.g., maintain a position while standing on 1 foot -weight shifting e.g., shifting weight to the stance leg while kicking a ball	•The test consists of 2 parts: performance scales and participation checklists for home and school. •Each activity is given a total score based on performance on several items/tasks. •Individual items/tasks are scored on a 0-3 scale. •The overall score was reported as a scaled score. •The subtests scores are reported as the number of items in the scale in which the child demonstrated poor performance and received a“0” or a “1.

**Table 3:** Measurements of the social skill tests.

Author (Year)	Test Name	Age Coverage	Test Description	Procedure
Holloway J.M. et al. (2018) [29]	The Social Skills Improvement System Rating Scales (SSIS)	3 to 18 years	- Social skills •communication, cooperation, assertion, responsibility, empathy, engagement, self-control - Problem behaviors •internalizing behaviors, externalizing behaviors, hyperactivity, bullying, autism signs such as stereotypical movements or difficulty transitioning during daily routines. -Academic competence (not used in Holloway’s study)	Parent/caregiver questionnaire with Likert scales
MacDonald M. et al. (2013) [28]				
MacDonald M. et al. (2014) [35]	Calibrated severity scores		•Mapped based on ADOS but independent of verbal IQ	Observation of ADOS sessions
Colombo-Dougovito A.M. et al. (2017) [34]	Social Responsiveness Scale (SRS)	4 to 18 years	•receptive, cognitive, expressive, motivation aspects of social behavior •autistic preoccupation	Parent-completed screening questionnaire
Hirata S. et al. (2014) [25]				
Travers B.G. et al. (2015) [30]				
Travers B.G. et al. (2013) [33]				
Bishop-Fitzpatrick L. et al. (2017) [31]	•Child Behavior Checklist/4–18 (CBCL)	4 to 18 years	•emotional, behavioral, social problems	Parent report with Likert scales
	The Vineland Adaptive Behavior Scales, 2nd Ed. (VABS-2)	Birth to 90 years	- Socialization domain •interpersonal relationships (only Mody uses this), leisure, coping skills	Parent/caregiver questionnaire
Pusponegoro H.D. et al. (2016) [22]				
MacDonald M. et al. (2013) [13]				
Mody M. et al. (2017) [26]				
Mody M. et al. (2017) [26]	Autism Diagnostic Observation Schedule (ADOS)	2 to 17 years	•communication •social interaction (Mody used only) •play/imagination, •restricted and/or repetitive behaviors	Assessment through observation of ADOS sessions
Hannant P. et al. (2016) [23]	Autism Diagnostic Observation Schedule -2nd Edition (ADOS-2)			
	Autism Diagnostic Interview-Revised (ADI-R)	18 months and above	•social and communication •repetitive stereotyped behaviors •sensory and motor skills •talents •challenging behaviors	Detailed semi-structured interview to gather evidence from an informant (parent, sibling or partner of an individual)
Craig F. et al. (2018) [24]	The Social Communication Questionnaire (SCQ)	Over 4 years, with a mental age over 2 years	Current and lifetime forms available •communication •reciprocal social interactions •restricted and repetitive behaviors and interests	Parent/caregiver questionnaire with 40 yes-or-no items
Dadger H. et al. (2017) [32]	Early Social Communication Scale (ESCS)	8 to 30 months	Nonverbal communication skills •initiating joint attention •responding to joint attention •initiating behavioral requests •responding to behavioral requests •initiating social interaction •responding to social interaction	Semi-structured assessment through observation of a videotaped session
Kim H. et al. (2016) [21]	Devereux Early Childhood Assessment (DECA)	2 to 5 years	•initiative •self-control •responses	After an observation period of at least 4 weeks, the DECA is completed by both a parent and a teacher
Hsu H.C. et al. (2004) [27]	Chinese Children Developmental Inventory (CCDI)	6 to 78 months	•expressive language •concept comprehension •social comprehension •personal social	

IQ, intelligence quotient.
